# Establishment and Comparison of Juvenile Female Mouse Models of Nonalcoholic Fatty Liver Disease and Nonalcoholic Steatohepatitis

**DOI:** 10.1155/2018/8929620

**Published:** 2018-08-07

**Authors:** Lishan Zhou, Deliang Liu, Zhiwei Wang, Hui Dong, Xiaohu Xu, Shunchang Zhou

**Affiliations:** ^1^Department of Integrated Traditional Chinese and Western Medicine, Wuhan Children's Hospital (Wuhan Maternal and Child Healthcare Hospital), Tongji Medical College, Huazhong University of Science and Technology, Wuhan, Hubei 430016, China; ^2^Endocrinology Department of Shenzhen Traditional Chinese Medicine Hospital, Shenzhen, Guangdong 518033, China; ^3^Endocrinology Department of Muping Traditional Chinese Medicine Hospital, Yantai, Shandong 264100, China; ^4^Institute of Integrated Traditional Chinese and Western Medicine, Tongji Hospital, Tongji Medical College, Huazhong University of Science and Technology, Wuhan, Hubei 430030, China; ^5^Department of Integrated Traditional Chinese and Western Medicine, Tongji Hospital, Tongji Medical College, Huazhong University of Science and Technology, Wuhan, Hubei 430030, China; ^6^Center of Experimental Animals, Tongji Medical College, Huazhong University of Science and Technology, Wuhan, Hubei 430030, China

## Abstract

Experimental research has successfully established an adult offspring animal model of nonalcoholic fatty liver disease (NAFLD), but the female offspring model of NAFLD in young age has not been well characterized yet. The aim of this study was to present a direct comparison of the maternal versus postweaning female juvenile NAFLD and nonalcoholic steatohepatitis (NASH) animal models. Four different female mouse models were established and compared using different high-fat diet feeding (HF) strategies in maternal mice and their offspring. The models were non-HF maternal mice and HF offspring with high-high fat (C/HHF), non-HF maternal mice and HF offspring with low-high fat (C/LHF), HF maternal mice and offspring both with high-high fat (HHF/HHF), and HF maternal mice and offspring both with low-high fat (LHF/LHF). A female control group (C/C) was also established. The offspring mice were raised to the age of 8 weeks and then euthanized. Blood glucose levels, lipid profiles, liver function, and triglycerides/total cholesterol contents were examined. Hepatic morphology and superoxide anion levels were evaluated. The nicotinamide-adenine dinucleotide phosphate activity and related regulatory subunits protein expression in the liver tissue were also determined. Our data demonstrated that offspring fat intake contributed to the successful establishment of NAFLD and maternal-offspring fat intake contributed to the successful establishment of NASH in juvenile female mice. Offspring high-fat exposure might be associated with the development of NAFLD and maternal high-fat exposure might be associated with the development of NASH in juvenile female offspring. Higher calories from a fat diet program (both in maternal and offspring) are more prone to inducing liver injury in offspring. In addition, the combination of the aforementioned two factors could aggravate this process. Moreover, oxidative stress was prominent in the juvenile female mouse model of NAFLD/NASH, and the mechanism might be related to the activation of liver NADPH oxidase.

## 1. Introduction

The prevalence of nonalcoholic fatty liver disease (NAFLD) is rapidly increasing with the global obesity epidemic [1, 2]. NAFLD encompasses a broad spectrum of liver disease, ranging from simple steatosis to nonalcoholic steatohepatitis (NASH) with fibrosis [3, 4]. Recently, NAFLD was common in children, which is a major cause for referral to pediatric gastroenterologists and hepatologists [5–7]. Accumulating evidence has proved that pediatric NAFLD and NASH are closely related to adult cirrhosis, hepatocellular carcinoma, and metabolic syndrome [8]. Therefore, research on nosogenesis and therapies to prevent and treat pediatric NAFLD is absolutely necessary.

Unfortunately, to date, there have been no effective treatments for children and adolescents with NAFLD [9]. Animal models representing NAFLD and NASH have been well documented in the search for effective anti-NAFLD drugs [10–13]. It is believed that exposure to a maternal high-fat diet (HFD) contributes to NAFLD progression in the offspring and has more detrimental effects on the offspring's liver than a simple postweaning HFD [14, 15]. However, younger offspring and female offspring remain debatable, and HFD composition is inconsistent among many of these models. Recently, a female animal model of juvenile NAFLD and NASH was established with an after weaning HFD without intrauterine HFD programs [16]. Therefore, the aim of this study was to present a direct comparison of the maternal versus postweaning female juvenile NAFLD and NASH animal models and to evaluate the effect of two different HFDs.

In addition, oxidative stress (OS) propels the progression of NAFLD to the end and is well validated to be the real “first hit” of a “two hit” hypothesis by a series of research studies over the past few years [8, 17, 18]. From previously reported data, different mechanisms between males and females may exist. Female juvenile NAFLD and NASH model animals presented hepatic OS, while male model animals presented hepatic inflammation [16]. Hence, our study also focuses on comparing the degree of OS and the change of nicotinamide-adenine dinucleotide phosphate (NADPH) activity and related protein expression in the liver between the maternal and the postweaning female juvenile NAFLD and NASH animal models.

## 2. Materials and Methods

### 2.1. Animals

This study used male (*n* = 10) and female (*n* = 30) C57BL6J mice, aged 56 to 63 days, obtained from the Institute of Laboratory Animal Sciences, Chinese Academy of Medical Sciences and Peking Union Medical College (certification number 11401300022814). The mice were maintained at an ambient temperature (22°C ± 1°C) with a 12 : 12 hour light-dark cycle and had free access to water and food (Barrier system, Center of Experimental Animals, Tongji Medical College, Huazhong University of Science and Technology; certification number 00132595). All experiments were approved by the animal ethics committee of Wuhan Medical and Health Center for Women and Children, Huazhong University of Science and Technology (number 2014010).

### 2.2. Diets

High-fat diets were purchased from Research Diets Inc. (New Brunswick, NJ, USA), including high HFD (HHF) D12492 in a blue color, 5.24 total kcal/gm (60% kcal and 34.9% g fat, 20% kcal and 26.2% g protein, and 20% kcal and 26.3% g carbohydrate) and low HFD (LHF) D12451 in a red color, 4.73 total kcal/gm (45% kcal and 24% g fat, 20% kcal and 24% g protein, and 35% kcal and 41% g carbohydrate). The Laboratory Animal Center of Tongji Medical College of Huazhong University of Science and Technology provided a standard diet (35% flour, 20% soy meal, 20% corn meal, 15.5% bran, 0.5% bean oil, 5% fish meal, 2.5% bone meal, 1% dusty yeast, and 0.5% salt).

### 2.3. Animal Modeling and Grouping

**Figure 1:**
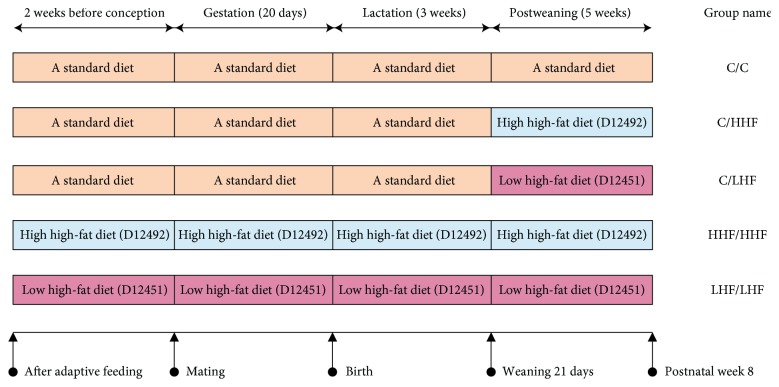
Animal modeling and grouping. Female maternal mice were fed either a standard diet (C) or different high-fat diets (HHF/LHF) 2 weeks before conception, during pregnancy and lactation. Female offspring mice were fed either a standard diet (C) or different high-fat diets (HHF/LHF) during postweaning up to 8 weeks of age. The numbers in parentheses indicate the type of high-fat diets.

After adaptive feeding with a standard diet for 1 week, all the female mice were randomized into the normal control group (*n* = 6) and four model groups (numbers 1 to 4, *n* = 6 in each). All the male mice were in the same randomization, with a ratio of 1 : 3 to the female mice (*n* = 2 in each group) for mating. All the male mice continued the standard diet. The female mice in the control group and the former two model groups also continued on the standard diet 2 weeks before conception and during gestation and lactation, whereas the female mice in the latter two model groups were fed relevant high-fat diets 2 weeks before conception and during gestation and lactation. Litter size was standardized to four pups to guarantee that no litter was nutritionally biased. After weaning, the female offspring mice in the control group were fed a standard diet. The female offspring mice in all four model groups were given relevant high-fat diets, generating five experimental groups: C/C (*n* = 8), C/HHF (*n* = 8), C/LHF (*n* = 8), HHF/HHF (*n* = 8), and LHF/LHF (*n* = 8), as illustrated in Figure 1.

### 2.4. Sampling

Based on the age of body maturation [19], all the offspring female mice were euthanized at the end of 8 weeks. Blood samples were obtained by orbital sinus puncture at the time of euthanization. The specimen serum was collected and stored at −20°C for analysis after centrifuging at 3000 revolutions per minute for 30 minutes at 4°C. The livers were quickly removed by laparotomy and flushed with normal saline on ice. Samples from the left outside lobe of the liver were fixed in a 4% paraformaldehyde solution for paraffin embedding. Samples from the left inside lobe of the liver were prepared for frozen sections. Samples from the right lobe and the caudate lobe of the liver were preserved at −80°C for analysis.

### 2.5. Oral Glucose Tolerance Test (OGTT)

Three days before euthanization, the offspring mice fasted for 12 hours. Afterward, 50% glucose was orally administered at a dose of 2 g/kg. Blood samples were collected from the tail veins at 0 (prior to glucose loading), 60, and 120 minutes (after glucose loading). Blood glucose levels were tested via the glucose-oxidase method using a glucose monitor (Accu-Chek Performa, Roche, Basel, Switzerland).

### 2.6. Biochemical Analysis

The serum levels of the triglycerides (TG), total cholesterol (TC), high-/low-density lipoprotein cholesterol (HDL-C/LDL-C), liver content of TG/TC, blood alanine aminotransferase (ALT), and aspartate aminotransferase (AST) were examined using commercial reagents (Jiancheng Bioengineering Institute, Nanjing, China).

### 2.7. Hepatic Histology

The paraffin slides were stained with hematoxylin and eosin (H&E) and observed under an optical microscope to assess the hepatocyte steatosis and cellular infiltrate, with sirius red observed under a polarizing microscope (Axio Scope.A1, Carl Zeiss, Oberkochen, Germany) to assess the collagen deposition of the liver. The following criteria were used for scoring hepatic histology. The Kleiner scoring system was used to evaluate the severity of NAFLD [20]. An activity score was generated by adding the individual scores for three features: steatosis (<5% = 0, 5%–33% = 1, 33%–66% = 2, and >66% = 3), ballooning (none = 0, few = 1, and prominent = 2), and lobular inflammation (none = 0, <2 foci = 1, 2–4 foci = 2, and >4 foci = 3). A score of less than 3 represents a mild nonalcoholic fatty liver, a score of 3 to 4 represents a moderate nonalcoholic fatty liver, and a score of 5 or more represents nonalcoholic steatohepatitis (NASH).

### 2.8. Measurement of Hepatic Superoxide Anion Levels

Serving as an oxidant-sensitive probe, dihydroethidium (DHE) is widely used for the measurement of reactive oxygen species (ROS). Ethidium and 2-hydroxyethidium, two products of DHE oxidation, bind to the nuclear DNA, thereby forming a strong red fluorescent complex [21, 22]. Frozen sections of the liver (6 *μ*m) were incubated with DHE (5 mmol/l, Beyotime Institute of Biotechnology, Shanghai, China) in a dark container at 37°C for 30 minutes [23] and then observed under an inverted microscope (IX51, Olympus, Tokyo, Japan).

### 2.9. Measurement of Liver NADPH Activity

Liver homogenate was lyzed in mammal tissue protein extraction reagent. The extracted protein was then supplemented with a protease inhibitor cocktail and phenylmethylsulfonyl fluoride (PMSF) (Sinopharm Chemical Reagent Co. Ltd., Shanghai, China). The samples were then centrifuged at 12,000 revolutions per minute for 15 minutes at 4°C. The supernatant was collected to quantify the protein concentration using a BCA protein assay kit (ASPEN Biotechnology Co. Ltd., Wuhan, China). Liver NADPH activity was measured using an NADPH activity quantification kit (Genmed Scientifics Inc., Shanghai, China).

### 2.10. Western Blotting Analysis

Liver-extracted proteins (40 *μ*g) were mixed with sample buffer, boiled for 5 minutes, and subjected to 8%, 10%, and 12% SDS-PAGE gel graded from maximum to minimum of protein molecular weight (120 volts, 60–90 min). Separated proteins on the gel were transferred to 0.45 *μ*m polyvinylidene fluoride membranes. The membranes were then blocked with 5% fat-free dry milk in TBST at room temperature for 1 hour, followed by incubation overnight with antibodies (p47^phox^, p22^phox^, and GAPDH) (Abcam, Hong Kong, China) (diluted 1 : 500, 1 : 1000, and 1 : 10000) at 4°C. The next day, after being washed with TBST three times, the membranes were incubated with HRP-goat anti-rabbit antibody (KPL Company, Hong Kong, China) diluted 1 : 10000 at room temperature for 30 minutes. Then, after being washed with TBST four times, immunoreactive proteins were detected using the chemiluminescence method (LiDE110, Canon, Tokyo, Japan). Finally, band densities were determined using AlphaEaseFC software and quantified as the ratio between the OD value of the target band to that of GAPDH.

### 2.11. Statistical Analysis

All data were analyzed using SPSS 19.0 statistical software. Statistical significance was assessed by one-way analysis of variance (ANOVA) following K-S normality. With homogeneity of variances, significance between different groups was determined using the least standard deviation (LSD) test; alternatively, the Games-Howell test was used. A probability of less than 0.05 was considered statistically significant.

## 3. Results

### 3.1. Comparison of Diet-Induced Fatty Liver among Juvenile Female Offspring in Four Models

#### 3.1.1. Histological Analysis

**Figure 2:**
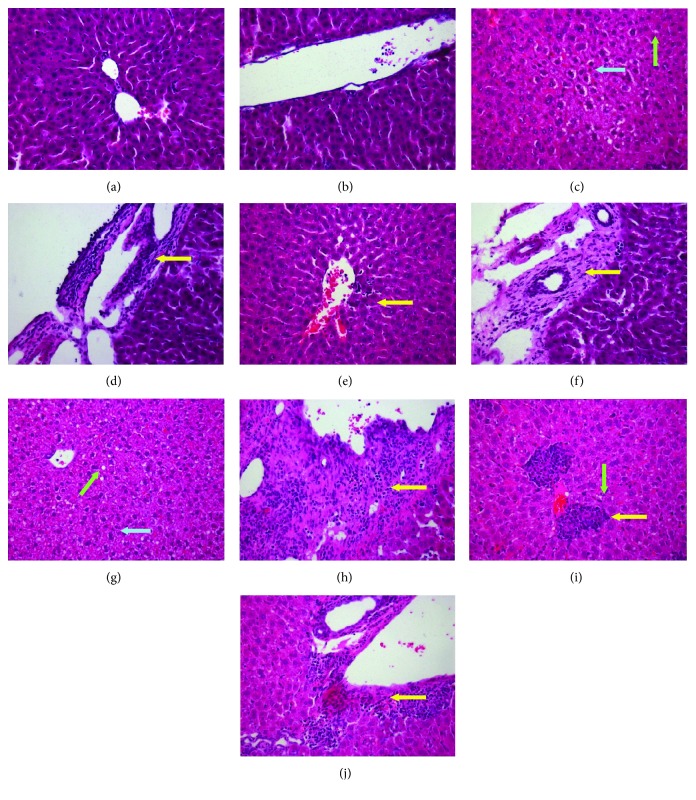
Representative histological images of mice hepatic tissue (hematoxylin and eosin staining). Original magnification: ×100. (a, b) C/C; (c, d) C/HHF; (e, f) C/LHF; (g, h) HHF/HHF; (i, j) LHF/LHF. Green arrows are pointing at the hepatocyte steatosis; blue arrows are pointing at the hepatocyte swelling; yellow arrows are pointing at the fibroplasia and inflammation in the portal area.

As shown in Figure 2, H&E-stained liver tissues of mice in all the model groups showed hepatocyte steatosis, fibroplasia, and inflammation in the portal area (type 2) instead of the perisinusoidal area (type 1) with lymphocytic infiltrate. However, the hepatic architecture was nearly normal. Ballooned hepatocytes were also observed in the HHF/HHF and LHF/LHF groups. The severity of NAFLD/NASH in the model mouse livers was assessed using the Kleiner scoring system [20], which allows the scoring of individual features, including steatosis, ballooned hepatocytes, and inflammation. As shown in Table 1, the Kleiner score of the mice in the C/C group was 0. The C/HHF mice achieved a score of 4, which indicates moderate NAFLD with evidence of inflammation, mild steatosis, and ballooned hepatocytes. The C/LHF mice achieved a score of 2.67, which indicates mild NAFLD with evidence of mild inflammation and steatosis and no ballooned hepatocytes. Both the HHF/HHF and LHF/LHF offspring presented with NASH, as indicated by scores of 6.33 and 5.66, respectively. In addition, inflammation, hepatocyte steatosis, and ballooned epatocytes in the offspring mice of the HHF/HHF group were more severe than those in the mice in the LHF/LHF group.

#### 3.1.2. Liver Function

**Figure 3:**
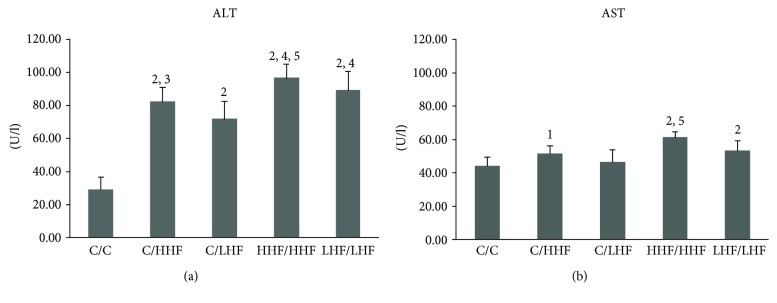
Liver function in offspring mice of different groups. Values are mean ± SD (*n* = 8). (a) ALT levels; (b) AST levels. ^1^*p* < 0.05, ^2^*p* < 0.01 versus C/C group; ^3^*p* < 0.05, ^4^*p* < 0.01 versus C/LHF group; ^5^*p* < 0.01 versus C/HHF group. ALT: alanine aminotransferase; AST: aspartate aminotransferase.

Compared to the C/C group, ALT levels significantly increased in all model groups (*p* < 0.01) and AST levels in all groups except C/LHF (*p* < 0.05, at least). The HHF/HHF group exhibited the highest ALT and AST levels (Figure 3).

#### 3.1.3. Liver TC and TG Contents

**Figure 4:**
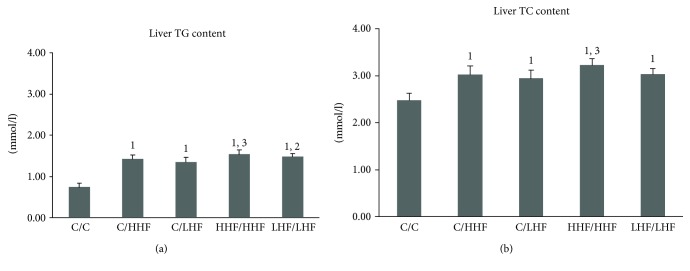
Liver TG and TC contents in offspring mice of different groups. Values are mean ± SD (*n* = 8). (a) Liver TG content levels; (b) liver TC content levels. ^1^*p* < 0.01 versus C/C group; ^2^*p* < 0.05, ^3^*p* < 0.01 versus C/LHF group. TG: triglyceride; TC: total cholesterol.

Compared to the C/C group, both liver TG and TC contents markedly increased in all model groups (*p* < 0.01). The HHF/HHF and LHF/LHF model mice presented a slightly higher liver TG content than the C/LHF model mice (*p* < 0.05, *p* < 0.01); only the HHF/HHF model mice presented a slightly higher liver TC content than the C/LHF model mice (*p* < 0.01) (Figure 4).

### 3.2. Comparison of Glucolipid Metabolism Disorders among Juvenile Female Offspring in Four Models

#### 3.2.1. Body Weight

**Figure 5:**
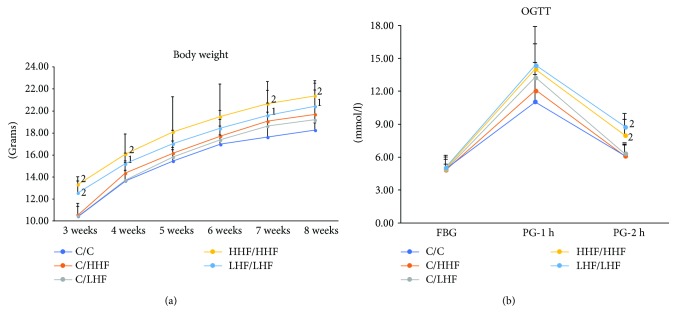
Body weight and OGTT in offspring mice of different groups. Values are mean ± SD (*n* = 8). (a) Body weight; (b) OGTT. ^1^*p* < 0.05, ^2^*p* < 0.01 versus C/C group. OGTT: oral glucose tolerance test; FBG: fasting blood glucose; PG-1 h: postprandial blood glucose at 1 hour after glucose loading; PG-2 h: postprandial blood glucose at 2 hours after glucose loading.

As shown in Figure 5, there were no significant differences in body weight (BW) among the C/C, C/HHF, and C/LHF mouse groups. Conversely, HHF/HHF and LHF/LHF groups showed a clear trend to an increase in BW as compared to the C/C group that achieved statistical significance (*p* < 0.01 and *p* < 0.05, resp.) at the ages of 3, 4, 7, and 8 weeks.

#### 3.2.2. OGTT

As shown in Figure 5, there were no differences in the fasting blood glucose (FBG) and postprandial blood glucose 1 hour after glucose loading (PG-1 h) levels among all the groups. However, compared to the C/C group, postprandial blood glucose 2 hours after glucose loading (PG-2 h) levels significantly increased in the HHF/HHF and LHF/LHF model groups (*p* < 0.01), which were the groups showing a significant overweight.

#### 3.2.3. Plasma Lipid Profiles

**Figure 6:**
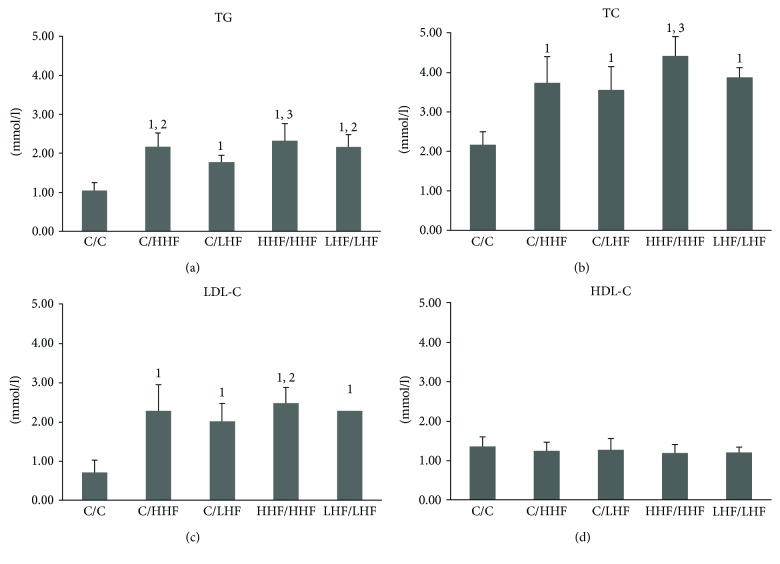
Plasma lipid profiles in offspring mice of different groups. Values are mean ± SD (*n* = 8). (a) TG levels; (b) TC levels; (c) LDL-C levels; (d) HDL-C levels. ^1^*p* < 0.01 versus C/C group; ^2^*p* < 0.05, ^3^*p* < 0.01 versus C/LHF group. TG: triglyceride; TC: total cholesterol; LDL-C: low-density lipoprotein cholesterol; HDL-C: high-density lipoprotein cholesterol.

As shown in Figure 6, the mice in all model groups exhibited a significant elevation in plasma TG, TC, and LDL-C levels, compared to the C/C group mice (*p* < 0.01). However, plasma HDL-C levels were similar in all of the groups. The C/HHF, HHF/HHF, and LHF/LHF model mice presented higher plasma TG levels than the C/LHF model mice (*p* < 0.05, *p* < 0.01); only the HHF/HHF model mice presented higher plasma TC and LDL-C levels than the C/LHF model mice (*p* < 0.05, *p* < 0.01).

### 3.3. Comparison of Liver Fibrosis and Oxidative Stress among Juvenile Female Offspring in Four Models

#### 3.3.1. Liver Collagen Deposition

**Figure 7:**
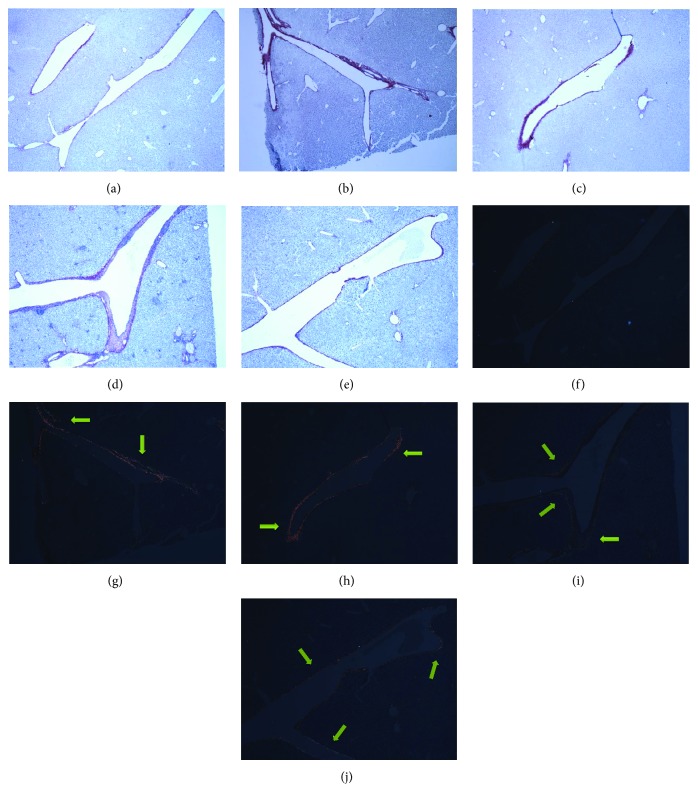
Representative images of sirius red staining in the portal area from the mice in different groups. Original magnification: ×50. (a, f) C/C; (b, g) C/HHF; (c, h) C/LHF; (d, i) HHF/HHF; (e, j) LHF/LHF. (a–e) Using optical microscope. (f–j) Using polarizing microscopy. Arrows are pointing at the collagen deposition.

To verify liver fibrosis, we observed collagen deposition in the portal and perisinusoidal areas using sirius red-stained liver sections in both the C/C group and all of the model groups (Figure 7). Collagen deposition mainly occurred in the portal area of the liver tissues in the mice in the C/HHF and C/LHF groups. It manifested as a bright red light under polarizing microscopy, indicating portal fibrosis with type I collagen deposition. Collagen was also deposited in the portal area of the liver tissues in the mice in the HHF/HHF and LHF/LHF groups. However, the type of collagen was different. Under microscopy, it manifested as a dim yellow light in the portal area of the liver tissues in the HHF/HHF group, indicating portal fibrosis with type IV collagen deposition. However, both bright red light and dim yellow light were observed in the LHF/LHF group, suggesting portal fibrosis with both types I and IV collagen deposition. In addition, thickening of the basement membrane was observed in the HHF/HHF group, indicating the most severe liver fibrosis of the four model groups.

#### 3.3.2. Hepatic Superoxide Anion Production

**Figure 8:**
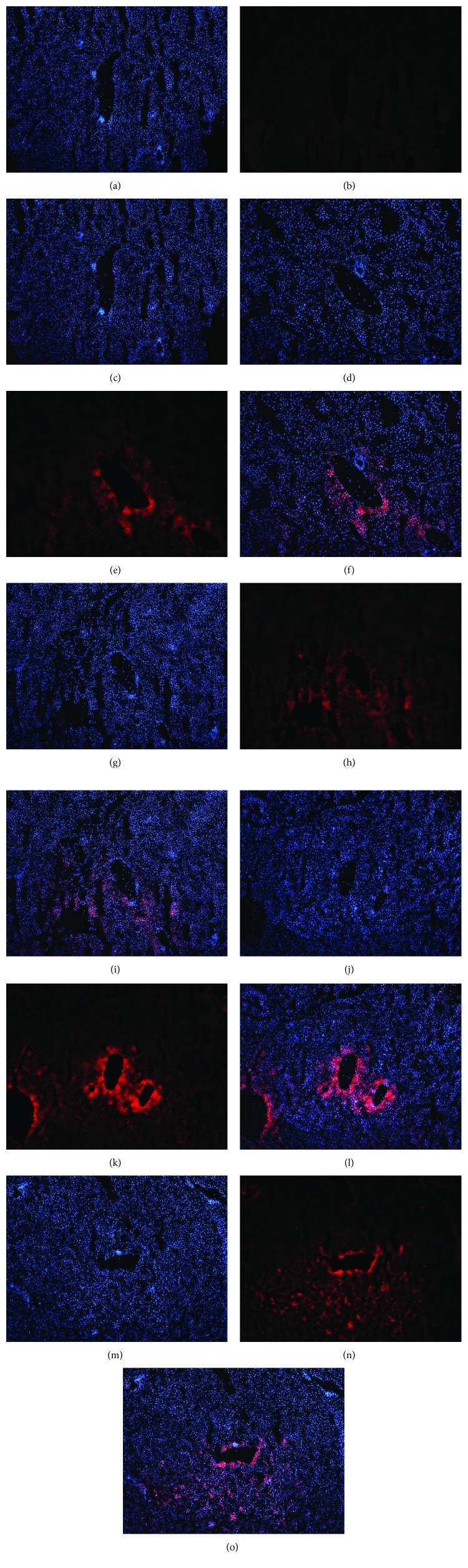
Representative images of dihydroethidium staining in the liver from the mice in different groups. Original magnification: ×40. (a, b, c) C/C; (d, e, f) C/HHF; (g, h, i) C/LHF; (j, k, l) HHF/HHF; (m, n, o) LHF/LHF. (a, d, g, j, m) Visualization of the nucleus in the liver using DAPI stains. (b, e, h, k, n) Visualization of ROS in the liver using DHE stains. (c, f, i, l, o) The superimposed photos of different groups.

As shown in Figure 8, compared to the control mice, a high level of DHE fluorescence was observed in the hepatic tissues in all of the model mice, indicating increased superoxide anion production. The HHF/HHF group presented the strongest red light in the liver tissue, indicating the most severe hepatic oxidative stress.

### 3.4. Comparison of Hepatic NADPH Activity and Involved Subunit Protein Expression among Juvenile Female Offspring in Four Models

#### 3.4.1. Hepatic NADPH Activity

**Figure 9:**
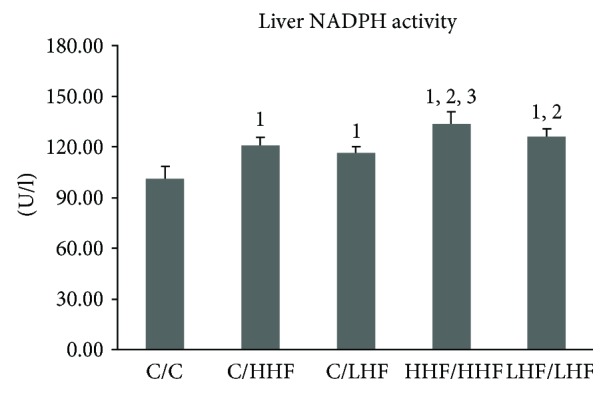
Hepatic NADPH activity in offspring mice of different groups. Values are mean ± SD (*n* = 8). ^1^*p* < 0.01 versus C/C group; ^2^*p* < 0.01 versus C/LHF group; ^3^*p* < 0.01 versus LHF/LHF group. NADPH: nicotinamide-adenine dinucleotide phosphate.

As shown in Figure 9, the activity of hepatic NADPH was much higher in the four groups of model mice than in the control mice (*p* < 0.01). The HHF/HHF and LHF/LHF model mice presented higher hepatic NADPH activity than the C/LHF model mice (*p* < 0.01), being the HHF/HHF group the highest.

#### 3.4.2. Hepatic p47^phox^ and p22^phox^ Protein Expression

**Figure 10:**
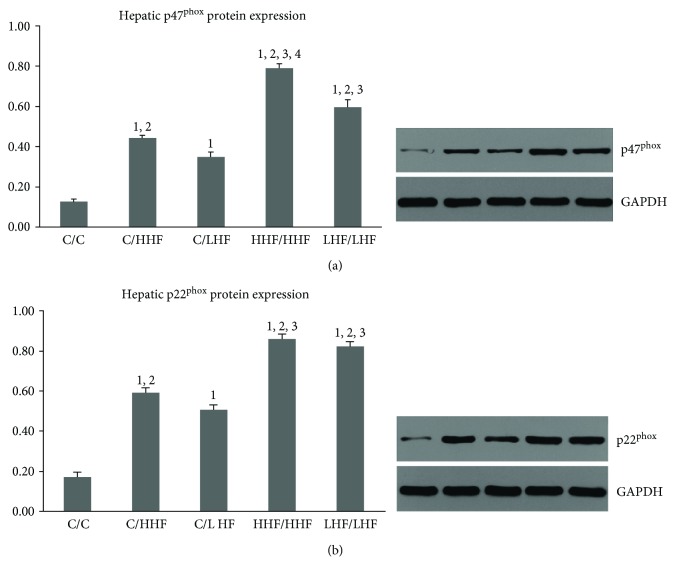
The protein expressions of hepatic p47^phox^ and p22^phox^ in offspring mice of different groups. Values are mean ± SD (*n* = 6). (a) The protein expression of hepatic p47^phox^; (b) the protein expression of hepatic p22^phox^. ^1^*p* < 0.01 versus C/C group; ^2^*p* < 0.01 versus C/LHF group; ^3^*p* < 0.01 versus C/HHF group; ^4^*p* < 0.01 versus LHF/LHF group.

As shown in Figure 10, hepatic p47^phox^ and p22^phox^ protein levels were significantly increased in the four mouse models compared to the control group mice (*p* < 0.01). Among all four models, the HHF/HHF group showed the highest expression of hepatic p47^phox^ protein (*p* < 0.01). However, both mice in the HHF/HHF and LHF/LHF groups showed the highest expression of hepatic p22^phox^ protein (*p* < 0.01).

## 4. Discussion

As mentioned in a previous study, in the development of pediatric NAFLD and NASH, the HFD exposure time can divide into two stages: exposure by the maternal method (prenatal, pregnancy, and lactation) and by the offspring method (postweaning) [14, 24–26]. Based on these two stages and different calorie formula diets (60% and 45% of calories from fat, resp.), we set up four maternal-offspring HFD mouse model groups: C/HHF, C/LHF, HHF/HHF, and LHF/LHF. Then, we compared the differences in NAFLD and NASH among the juvenile female offspring mice.

In our study, the young female offspring mouse model of NAFLD was successfully established with a HFD in offspring program, while the model of NASH was successfully established with a HFD in maternal-offspring program. First, Kleiner score analysis was performed to analyze the phenotype of fatty liver. The score showed that the C/HHF and C/LHF groups predisposed the juvenile female offspring mice to develop NAFLD, the benign form of hepatic steatosis. However, the HHF/HHF and LHF/LHF groups caused a progressive form of steatohepatitis along with hepatocyte injury and inflammation. The results also confirmed that a distinct histological pattern in pediatric NAFLD is portal area damage (type 2) [20, 27, 28], in contrast to the predominant damage in the perisinusoidal area (type 1) of adult NAFLD. Second, the elevated plasma ALT and AST (except for the C/LHF group) levels indicated hepatocyte injury. The HHF/HHF group showed the most severe liver dysfunction. Third, the increased liver TG and TC contents indicated fat accumulation in the liver tissue. Both maternal-offspring HFD model groups showed a higher liver TG content. And the HHF/HHF young female offspring mice showed the highest liver TC content.

The juvenile female offspring mice only in two maternal-offspring HFD model groups were characterized by increase of body weight and an impairment of oral glucose tolerance and no difference between them. However, the juvenile female offspring mice in all of the model groups manifested the characteristics of hyperlipidemia. Liver TG and TC contents reflect plasma TG and TC levels in all the experimental groups. The plasma LDL-C levels of the HHF/HHF young female offspring mice were higher than those of the other three young female offspring mouse groups (C/HHF, C/LHF, and LHF/LHF).

Next, we evaluated liver fibrosis using sirius red staining. Fibroplasia was also observed in the portal area. The outcome proved that maternal HFD might be positively related to the extent and variety of collagen deposition. ROS was then validated using OS measures. Excessive ROS generated by OS induces oxidative damage via peroxidation of the biomacromolecule and interferes with hepatic cell signal transduction via serving as a second messenger [29]. To evaluate superoxide anion production, liver DHE staining was performed. The appearance of superoxide can change DHE into ethidium bromide, which binds to DNA and exhibits red fluorescence in the nucleus [30–32]. In our study, a massive increase in superoxide anion production was identified in all of the young model female offspring mice. Meanwhile, the HHF/HHF group was superior to other three model groups in enhancing oxidative stress.

Because OS activation was observed in our four juvenile female model mice, we performed studies in an effort to elucidate the potential mechanism. One liver enzyme indicating OS progression is NADPH oxidase from which ROS is mostly derived [29, 33, 34]. Accordingly, we first examined the hepatic NADPH activity and the protein expressions of its major two regulatory submits [33–35], p47^phox^ and p22^phox^. The result showed that NADPH activity was significantly elevated in all of the model groups. And the HHF/HHF model was the highest. Both p47^phox^ and p22^phox^ were concomitantly invoked. Two maternal-offspring HFD model groups showed a higher liver p47^phox^ and p22^phox^ protein expressions. And the HHF/HHF group also showed a much higher expression of liver p47^phox^.

In the end, we compared the same parameters between our present results of the C/HHF group and the results of the corresponding group (the female HFHC group). According to 16, a fructose-enriched HF-diet (D12331) (HFHC diet) was chosen, and four diet experimental checkpoints were designed. Marin et al. stated that a total of 8 weeks (equals to 11 weeks age) is the time required for the onset of NASH [36]. Although the mice presented a mixed macro-microvesicular steatosis and fibrosis after 8 weeks of HFHC diet, no significant alterations in TC, LDL-C, and ALT at that experimental checkpoints [16] were observed. In comparison with 16, the main discrepancy of our research was the diet. In the absence of fructose, compared with D12331 (5.56 total kcal/gm, 58% kcal and 35.8% g fat, 16.4% kcal and 23.0% g protein, and 25.5% kcal and 35.5% g carbohydrate), D12492 contains the same fat content, lower carbohydrate content, and higher protein content. High-fructose diet in adult rats causes obvious change on metabolism of serum lipids and lipid accumulation in the liver in comparison with HFD [37]. High-carbohydrate diet had more potent liver lipogenic and inflammatory effects in comparison with HFD [38]. High-protein diet prevents and reverse hepatic steatosis independently of fat and carbohydrate intake more efficiently [39]. Therefore, the HFHC diet should lead to more severe liver injury which opposed to the results of their and our works. However, all of these studies did not compare the two diets directly in setting up the juvenile female offspring mice model of NAFLD/NASH. The diet selection for modeling needs further study in the following work.

In summary, we concluded that offspring fat (both calorie formula diets) intake contributed to the successful establishment of NAFLD and maternal-offspring fat (both calorie formula diets) intake contributed to the successful establishment of NASH in the juvenile female mouse model. Both the maternal high-fat exposure and higher calories from fat diet program might be associated with the severity of the progress of juvenile female offspring NAFLD. And the aforementioned maternal high-fat exposure factor is more prone to inducing liver injury in offspring. In addition, the combination of the aforementioned two factors could aggravate this process. Moreover, OS was prominent in the juvenile female mouse model of NAFLD/NASH and the mechanism might be related to the activation of the liver NADPH oxidase. Nevertheless, due to the short duration of HF feeding in the present research, the progression of the disease needs further study in the following work.

## Figures and Tables

**Table 1:** Assessment of NAFLD/NASH severity in offspring livers.

Group	Steatosis	Ballooning	Inflammation	Activity score	Indication
C/C	0	0	0	0	Normal
C/HHF	0.67	0.33	3	4	NAFLD
C/LHF	1	0	1.67	2.67	NAFLD
HHF/HHF	2.33	1.33	2.67	6.33	NASH
LHF/LHF	2	1.33	2.33	5.66	NASH

Values are mean (*n* = 6). The Kleiner scoring system [20] and the average score for each histological characteristic in each group were used.
